# Meta-Analysis of Two Human RNA-seq Datasets to Determine Periodontitis Diagnostic Biomarkers and Drug Target Candidates

**DOI:** 10.3390/ijms23105580

**Published:** 2022-05-17

**Authors:** Carlos Moreno, Ellie Bybee, Claudia M. Tellez Freitas, Brett E. Pickett, K. Scott Weber

**Affiliations:** 1 Department of Microbiology and Molecular Biology, Brigham Young University, Provo, UT 84602, USA; carlosmoreno943@gmail.com (C.M.); ellie@bybeemail.com (E.B.); brett_pickett@byu.edu (B.E.P.); 2 College of Dental Medicine, South Jordan Campus, Roseman University of Health Sciences, South Jordan, UT 84095, USA; cfreitas@roseman.edu

**Keywords:** periodontitis, RNA-seq, diagnostic, biomarker, chronic, inflammation, drug, target, SPIA, gingiva

## Abstract

Periodontitis is a chronic inflammatory oral disease that affects approximately 42% of adults 30 years of age or older in the United States. In response to microbial dysbiosis within the periodontal pockets surrounding teeth, the host immune system generates an inflammatory environment in which soft tissue and alveolar bone destruction occur. The objective of this study was to identify diagnostic biomarkers and the mechanistic drivers of inflammation in periodontitis to identify drugs that may be repurposed to treat chronic inflammation. A meta-analysis comprised of two independent RNA-seq datasets was performed. RNA-seq analysis, signal pathway impact analysis, protein-protein interaction analysis, and drug target analysis were performed to identify the critical pathways and key players that initiate inflammation in periodontitis as well as to predict potential drug targets. Seventy-eight differentially expressed genes, 10 significantly impacted signaling pathways, and 10 hub proteins in periodontal gingival tissue were identified. The top 10 drugs that may be repurposed for treating periodontitis were then predicted from the gene expression and pathway data. The efficacy of these drugs in treating periodontitis has yet to be investigated. However, this analysis indicates that these drugs may serve as potential therapeutics to treat inflammation in gingival tissue affected by periodontitis.

## 1. Introduction

Periodontitis is a chronic inflammatory oral disease that affects approximately 42% of adults 30 years of age or older in the United States [1]. In 2016, periodontitis was determined to be the eleventh most common health condition globally [2], and in 2018 the direct and indirect economic burdens of periodontal disease within the U.S. were estimated to be about $154.06 billion and about €158.64B in Europe (sum of 32 European countries) [3]. Periodontitis is characterized by the recession of gums, alveolar bone loss, destruction of periodontal ligaments, and tooth decay in addition to swollen and bleeding gums. The cause of periodontitis is microbial dysbiosis within the periodontal pockets surrounding teeth, typically a result of improper oral hygiene. Interactions between pathogenic bacteria (primarily gram-negative *Porphyromonas gingivalis*, *Tannerella forsythia*, and *Treponema denticola* [4,5]) and the host immune system initiate an inflammatory environment that leads to the observed pathology [6,7]. If left untreated, the continued destruction of periodontal ligaments and alveolar bone may result in reduced tooth support and, eventually, tooth loss [8]. Although the prevention of this disease is possible through proper oral hygiene practices, such as brushing and flossing teeth daily, periodontal disease remains an important health issue globally due to its high prevalence and high economic burden [9,10].

Medical interventions that are required during severe periodontitis include the deep cleaning of tooth root surfaces to remove bacterial biofilms, antibiotics to kill pathogenic bacteria that are present, and corrective surgeries [11]. Medications directed at treating bacterial dysbiosis, such as antibiotics, just address a portion of the problem in periodontitis as they only reduce or temporarily reduce the bacterial burden associated with periodontitis [12]. In addition, the host immune response plays a critical role in the production of inflammatory mediators that results in the observed soft tissue damage [8]. Examples of inflammatory mediators are proteolytic enzymes and cytokines, which promote the differentiation of osteoclasts and are driving factors for soft tissue destruction in periodontal disease [8,13,14,15]. In a recent study, Reis et al. observed increased levels of inflammatory cytokines in periodontitis vs. healthy sites (pg/site), including interleukin 6 (IL-6) (0.13 vs 0.00), tumor necrosis factor-alpha (TNF-α) (0.06 vs. 0.01), interleukin 1α (IL-1α) (72.03 vs. 11.55), and interleukin 1β (IL-1β) (0.57 vs. 0.01) in the gingival crevicular fluid (GCF) of patients with chronic periodontitis who did not have any other underlying chronic systemic disorder [16]. Increased levels of pro-inflammatory C-reactive protein (80% increase) and fibrinogen (1.45 mg/L in generalized periodontitis and 1.30 mg/L in localized periodontitis versus 0.90 mg/L in healthy controls) have also been measured in periodontitis patients [17,18,19]. IL-1 and TNF-α can stimulate bone resorption by promoting the differentiation of osteoclasts in vitro [20]. Studies have shown that CD4+ helper T cells (Th), which release cytokines that recruit and activate other immune cells, play a major role in alveolar bone destruction during periodontitis [21,22]. Evidence suggests that T helper 17 (Th17) cells are drivers of periodontitis pathogenesis and that they are recruited to periodontal lesions by IL-6 and interleukin 23 (IL-23) [23]. T cells and granulocytes, such as neutrophils, make up more than 70% of immune cells present in periodontitis lesions [24]. The severity of periodontitis has been shown to correlate with the number of neutrophils that are present as well as the collagenase activity of these cells [24,25,26].

The impact of periodontitis on other systemic conditions highlights the importance of treating this illness and reducing chronic inflammation. Periodontitis has been associated with systemic diseases and disorders, such as adverse pregnancy outcomes, cardiovascular disease, pulmonary disease, rheumatoid arthritis, inflammatory bowel disease (IBD), and type 2 diabetes mellitus [27,28,29]. Studies in mice have demonstrated that microbial dysbiosis in periodontitis contributes to gut microbial dysbiosis and inflammation by ectopic colonization of pathogenic oral bacteria in the gut, which promotes inflammation in the colon [30,31,32]. A study conducted by Kitamoto et al. demonstrated how periodontitis could aggravate intestinal inflammation in mice with experimental colitis [31]. They observed that the increased colitis was associated with increased levels of Th17 and Th1 cells, as well as interleukin 17 A (IL-17A) and interferon-gamma (IFN-γ), in the colonic mucosa of mice that have experimentally induced periodontitis when compared to control animals. They also provide evidence that Th17 effector cells first arise in the oral cavity and then migrate to the colonic mucosa, where they contribute to the inflammation in the gut of the experimental colitis mice [31].

The development of RNA sequencing (RNA-seq) technology and bioinformatic tools have made it possible to investigate the changes in gene expression between healthy and diseased individuals to better elucidate the underlying mechanisms of observed pathologies. Transcriptomic analysis has also been used to identify biomarkers in cancer, chronic inflammatory diseases, and infectious diseases [33,34,35]. The public availability of transcriptomic datasets in the NCBI Gene Expression Omnibus (GEO) database enables researchers to perform meta-analyses on two or more datasets at a time [34,36,37]. The processing of multiple datasets as part of a meta-analysis increases the number of samples in order to achieve higher statistical power and reduce biases that may be present in individual datasets [38]. The lack of large-scale knowledge on the underlying transcriptomic mechanisms in periodontitis impedes the ability to effectively treat this disease.

Specifically, the underlying mechanistic drivers of inflammation in gingival epithelial cells and/or resident and recruited immune cells may serve as potential drug targets to mitigate the inflammatory response and consequently reduce tissue destruction in periodontitis. The objective of this study was to identify relevant inflammatory markers and potential drug targets that could be modulated to reduce the inflammatory response in human periodontitis. To identify these potential inflammatory markers and targets, RNA analysis [39,40,41], signaling pathway enrichment analysis (SPIA) [42], protein-protein interactions network [43,44,45], and drug target analysis [46,47] were used on two independent RNA-seq datasets. Here, the identification of 22 diagnostic biomarker candidates and 10 potential drug targets that may serve as potential therapeutics to reduce inflammation and tissue destruction in periodontitis are reported.

## 2. Results

### 2.1. RNA-seq Identification of Differentially Expressed Genes in Periodontal Gingival Epithelial Cells

**Figure 1:**
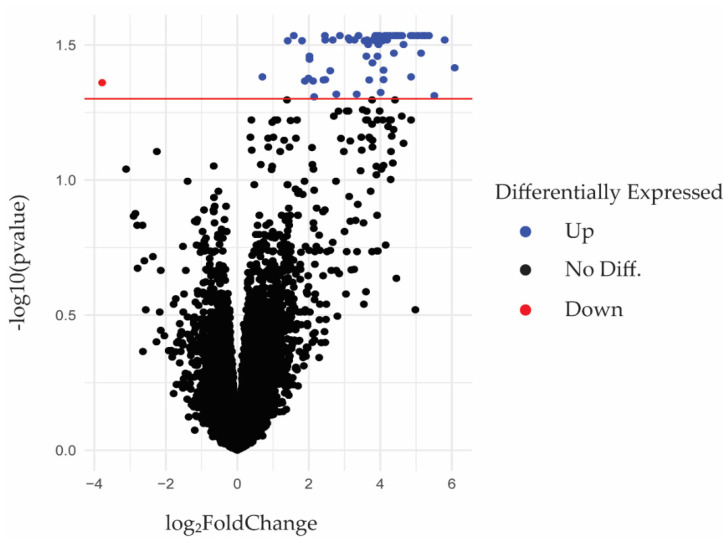
Volcano Plot of all differentially expressed genes (DEGs). The *x*-axis shows the log_2_ fold-change value for each gene, with positive and negative values representing up- and downregulation, respectively. The *y*-axis shows the −log_10_ adjusted *p*-value for each gene, with more significant values located higher on the *y*-axis. Base log_2_ and log_10_ were used on the *x*- and *y*-axis, respectively, to help visualize DEGs with very large or small fold changes and adjusted *p*-values. Significant DEGs are labeled as either red (downregulated genes; Down) or blue (upregulated genes; Up) dots and were determined according to the criteria adjusted FDR-adjusted *p*-value < 0.05. The horizontal red line shows the 0.05 adjusted *p*-value cutoff. All fold-change values were considered if the adjusted *p*-value was below 0.05. Seventy-seven DEGs were upregulated, and one gene (C1orf68) was downregulated in periodontal disease gingiva.

Samples from two separate human periodontitis RNA-seq datasets, which are publicly available in the NCBI Gene Expression Omnibus (GEO) database, were processed (Table 1). Our meta-analysis detected a total of 15,699 genes (Supplementary Table S1), with 78 of these being differentially expressed genes (DEGs) at our defined threshold for statistical significance (FDR-corrected *p*-value < 0.05), without regard for the magnitude of fold-change values (Figure 1). A comparison of differentially expressed genes (DEGs) between the current study and the previously published study by Kim et al. identified that approximately half (42) of the significant DEGs from the current study overlapped with the results from the prior work (Supplementary Figure S1) [48]. The study by Kim et al. used a different filtering strategy (cutoffs at adjusted *p*-value < 0.05 and a log_2_ fold-change value > ±2 compared to only an adjusted *p*-value < 0.05 cutoff). In addition, the data processing pipeline for the previously published study did not incorporate the same algorithms that were used in the current meta-analysis [48]. The number of DEGs overlapping between the current meta-analysis and the prior published study may be a result of differences in data processing and tissue sample processing between the two studies used in the meta-analysis. This phenomenon has been observed in previous studies [49]. An independent analysis of the RNA-seq data analyzed by Kim et al. using the ARMOR workflow was not performed since the data were pooled into one periodontitis SRA file and one healthy SRA file, which does not allow statistical analysis to be performed. No comparison of DEGs was made between the current study and the unpublished RNA-seq dataset (GSE173082) since no results or analysis were published.

Fifty-two of the 78 significant DEGs in our current study were immunoglobulin domains, and four DEGs were predicted as transcribed pseudogenes (Supplementary Table S2). The remaining 22 of the 78 significant DEGs included Bone Morphogenic Protein 6 (BMP6), Complement C3d Receptor 2 (CR2), Interferon Regulatory Factor 4 (IRF4), and others (Table 2). These genes function in gene transcription, metabolite transport, toll-like receptor signaling, chemokine secretion, inflammation, and endoplasmic reticulum stress. All but one of these 22 significant DEGs were upregulated in the gingiva of periodontal disease patients, with the exception being the downregulation of chromosome 1 open reading frame 68 (C1orf68), which had a log_2_ fold change (log_2_FC) of −3.78. 

### 2.2. Signaling Pathway Impact Analysis Identified 10 Significantly Impacted Pathways

The Signaling Pathway Impact Analysis (SPIA) algorithm was used to determine whether any known intracellular signaling pathways were enriched in DEGs. This robust approach uses a permutation-based analysis to generate a null distribution for each pathway, which yielded 10 pathways that were significantly affected in periodontitis gingival tissues (Table 3). Several relevant pathways, including “osteoclast differentiation” and “the innate immune system”, were observed, as well as several more generic pathways such as “cytokine–cytokine receptor interaction” and “leukocyte transendothelial migration”.

### 2.3. Drug Target Analysis Identified 500 Drugs That May Be Repurposed to Treat Periodontitis 

The next analysis step consisted of determining whether any of the affected signaling pathways contained proteins that are known targets of existing small molecules, monoclonal antibodies, and/or peptides that could be repurposed as potential therapeutics. Using the output from SPIA, 335 proteins in significant pathways were identified that are targets for 500 known drugs (Supplementary Table S2). It was observed that several drugs targeted known inflammatory mediators such as interleukins and toll-like receptors.

### 2.4. Protein-Protein Interaction Identification of Candidate Drug Targets against Top 10 Hub Proteins

**Figure 2:**
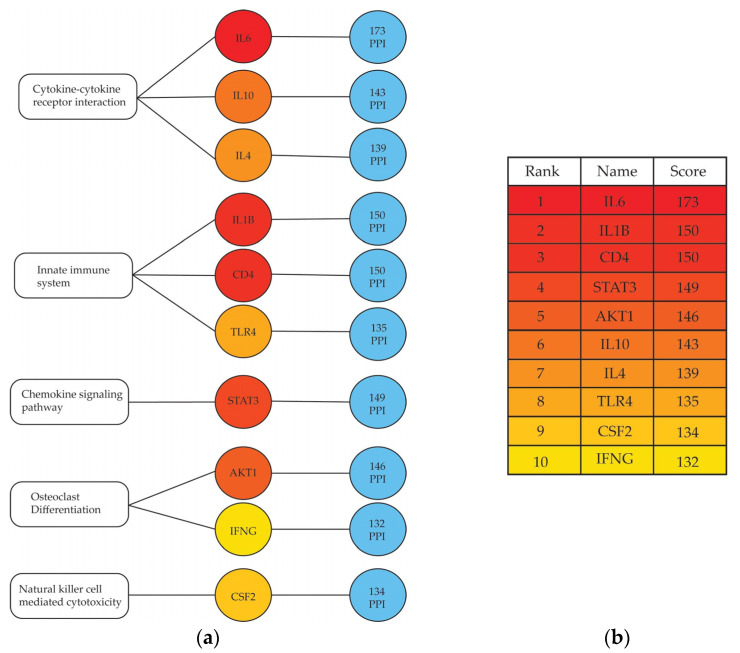
Top 10 hub proteins and their PPI rankings by degrees method. The 10 most-connected DEGs were identified as central hubs using cytoHubba based on their degrees of interactions with other genes/proteins. (**a**) Hub genes mapped back to five significantly impacted pathways, and the number of protein–protein interactions (PPI) for each are listed in blue circles. The proteins and their rank-by-degree scores are represented by a red-yellow color gradient. Red indicates the highest degree score, orange is intermediate, and yellow indicates the lowest; (**b**) Table of hub genes ranked by their degree score (PPI) using the same color gradient.

An unbiased approach was then applied to identify the drug targets that would be most likely to reverse the observed signaling pathway phenotype. Such a therapeutic approach could reduce or reverse some of the clinical signs and symptoms associated with the disease phenotype. To do so, the protein–protein interaction (PPI) network of drug targets that mapped back to all the statistically significant signaling pathways identified as playing a role in periodontitis gingival tissue were visualized. The initial PPI network, which was constructed using the online STRING database, consisted of 7462 edges and 304 nodes. CytoHubba was then used to reduce this initial network to the top 10 “central hub” proteins based on degrees (i.e., number of interacting neighbors) (Figure 2). 

Out of 500 drug candidates identified, 10 drugs that targeted the central hub proteins were identified as top candidates since they would be most likely to reverse the observed periodontitis phenotype (Table 4). A drug that targeted IL17RAwas also included. It was not one of the hub genes, but it was one of the top drug targets identified in the analysis, and its production by Th17 cells has been associated with bone resorption in periodontitis. This drug repurposing analysis also identified the IL-6 receptor (IL6R) and IL17RA proteins as targets for the FDA-approved drugs Satralizumab and Brodalumab that are approved to treat autoimmune diseases such as neuromyelitis optica spectrum disorder (NMOSD) and severe plaque psoriasis, respectively. 

## 3. Discussion

The aim of this study was to identify inflammatory biomarkers and the mechanistic drivers of inflammation, as well as to predict potential therapeutics for various aspects of periodontal disease. For this purpose, two independent RNA-seq datasets were retrieved from NCBI Gene Expression Omnibus (GEO) for RNA-seq meta-analysis. Dataset GSE80715 was previously analyzed in a published study that identified novel gene expression and splicing patterns in periodontitis gingival biopsies [48], whereas study GSE173082 is deposited in the GEO database but has not been published. RNA-seq meta-analysis identified 78 significant DEGs (FDR < 0.05) with 22 genes functioning in gene transcription, metabolite transport, toll-like receptor signaling, chemokine secretion, and endoplasmic reticulum stress. These 22 genes represent the top candidates as biomarkers to diagnose periodontitis, with 11 of these potential biomarkers found either extracellularly or on the surface of host cells (MZB1, MERTK, SCAMP5, C7, CR2, SMPDL3B, SLC17A9, BMP6, ST6GAL1, C1orf68, and LAX1). Future experiments could be designed to optimize a flow cytometry approach that could quantify these surface protein biomarkers as part of a periodontal disease diagnosis. The remaining 11 proteins (DERL, TENT5C, IRF4, SPAG4, XBP1, ANKRD44, NUGGC, FKBP11, PIM2, ENTPD7, and SEL1L3) are localized within the cytosol, Golgi apparatus, endoplasmic reticulum, or the nucleus of host cells. In the latter case, RT-qPCR may be useful in diagnosing severe periodontitis by measuring the transcripts of these genes from material collected from the affected site(s).

The signaling pathway impact analysis provided a higher level of analysis of how these differentially expressed genes contribute to the pathogenesis of periodontitis. For instance, the osteoclast differentiation pathway was significantly impacted and activated in periodontal gingival tissue. This is noteworthy since alveolar bone loss is a characteristic of severe periodontitis. Bone-resorbing osteoclasts work in conjunction with bone-forming osteoblasts during bone remodeling through cell–cell interactions and the secretion of signaling proteins (e.g., TNF superfamily member 11, or RANKL, and bone morphogenic protein 2, BMP2) to influence the activation and differentiation of each other [50,51]. Dysregulation in osteoclast or osteoblast activity can lead to excessive bone resorption (osteoporosis) or formation (osteopetrosis), respectively [52]. Overexpression of RANKL, osteoprotegerin (OPG), and macrophage colony-stimulating factor (M-CSF) can lead to excessive osteoclast activity [53]. Inflammatory cytokines and hormones, such as IL-1α/β, TNF-α, IL-6, IL-17, and Prostaglandin E2 (PGE2), may also promote osteoclast activity [53]. In a ligature-induced periodontitis rat model, mRNA levels of inflammatory cytokines such as IL-6, IL-1β, TNF-α, RANKL, and OPG were increased within the first week of inducing the disease [54]. However, this study found that mRNA levels of these cytokines were not significantly different two weeks post-induction [54]. This coincided with significant bone resorption up to two weeks post-induction [54]. The synergistic effects of T helper cells, B cells, macrophages, and neutrophils may also stimulate osteoclast activity during periodontitis [54].

Using transcriptomics to identify central hubs in protein–protein interaction networks is a novel approach in the field of periodontitis. The best 10 scoring results from this analysis included proteins such as IL-6, toll-like receptor 4 (TLR4), tumor growth factor-beta (TGF-β), and others. IL-6 and IL-1β are pro-inflammatory cytokines that are associated with chronic inflammation, periodontitis, and osteoclast bone resorption [20,23,53,54,55]. They were both identified as hub genes in the gingiva of periodontitis patients, making them potential candidates for therapeutic targets in treating inflammation in periodontitis. Interestingly, CD4 (cluster of differentiation 4) was found to be another hub gene. CD4 is a co-receptor/surface marker found on T helper cells, and it can also be found on the surface of macrophages, B cells, neutrophils, eosinophils, and mast cells [56]. Although there are currently no drugs that target CD4 to reduce inflammation or the function of CD4+ T cells, the identification of CD4 as a hub gene marks the importance of T helper cells in the inflammatory response during periodontitis. Signal transducer and activator of transcription 3 (STAT3), a transcription factor belonging to the STAT protein family, and AKT Serine/Threonine Kinase 1 (AKT1), a protein kinase that regulates cell growth and apoptosis [57,58,59] and acts as a signaling enzyme within the PI3 kinase signaling pathway [60], were also identified as hub genes. STAT3 plays important roles in the differentiation and function of several immune cells, including: dendritic cells [61,62,63,64], neutrophils [65], B cells [66], Th17 cells via IL-6 signaling [67,68], T follicular helper cells [69,70], and CD8+ cytotoxic T cells [65,71,72]. PI3K/AKT signaling promotes macrophage polarization [73] and T cell development and function [74,75]. This signaling pathway also regulates cell survival and glucose metabolism [76]. Interleukin 10 (IL-10), interleukin 4 (IL-4), and IFN-γ are other cytokines identified as hub genes with differing roles in inflammation. IL-10 is an anti-inflammatory cytokine that plays a protective role in mucosal surfaces against hyperinflammation, and the inhibition of IL-10 signaling has been found to promote the onset of colitis and irritable bowel syndrome (IBD) [77]. IL-4 is expressed by several lymphoid and myeloid cells, including T cells (primarily Th2), natural killer (NK) cells, eosinophils, basophils, and mast cells [78,79]. It plays important roles as a growth factor for B cells [80], IgE class switching in B cells [81], Th2 differentiation and response [82], and tissue repair [80]. IFN-γ is a pleiotropic cytokine that is primarily secreted by T cells and NK cells, and it plays major roles in the priming and activation of innate and adaptive immune cells, including dendritic cells and macrophages, as well as other NK cells and CD4+ and CD8+ T cells [83,84]. Lastly, TLR4 is a pattern recognition receptor that recognizes lipopolysaccharide (LPS) found on the outer membrane of gram-negative bacteria. Evidence also suggests that fragments of hyaluronan released during tissue damage may bind to TLR4 and initiate an inflammatory response [85]. Since gram-negative bacteria, such as *P. gingivalis*, are major contributors to microbial dysbiosis and inflammation in periodontitis, inhibiting the LPS-induced activation of TLR4 may be a potential mechanism to reduce inflammation in periodontitis.

When identifying the top 10 drug targets to treat inflammation in periodontitis, drugs that target hub genes and those drugs which have been FDA-approved were prioritized. Although one prior clinical study has evaluated the post-surgical use of systemic Doxycycline, we are not aware of other studies that have sought to investigate potential therapeutic treatments for periodontal disease. All of the drugs that are reported have been investigated for at least one indication, and some of them are approved for the treatment of chronic inflammatory (Satralizumab, Interferon beta-1b, Brodalumab), autoimmune (Satralizumab, Interferon beta-1b, Brodalumab), and dysregulated bone resorption diseases (Denosumab). Seven of the 10 drug candidates are monoclonal antibodies used to inhibit the signaling pathways of inflammatory cytokines. Satralizumab, Denosumab, and Brodalumab (Siliq) are monoclonal antibodies FDA-approved to treat anti-aquaporin-4 (AQP4) antibody-positive neuromyelitis optica spectrum disorder (NMOSD) [86,87], postmenopausal osteoporosis [88], and moderate to severe plaque psoriasis in adult patients [89], respectively. Cases of medication-related osteonecrosis of the jaw (MRONJ) were reported in clinical trials investigating the safety and efficacy of denosumab in treating metastatic bone cancer, multiple myeloma, and post-menopausal osteoporosis [90,91,92]. Multiple studies report a low incidence of MRONJ in osteoporosis patients treated with Denosumab [92,93,94]. However, the dosage of Denosumab for the treatment of periodontitis must be carefully considered, and the development of MRONJ monitored closely. Risk factors for MRONJ caused by bisphosphonates, anti-angiogenic drugs, and Denosumab, include invasive dental procedures (i.e., tooth extractions or other procedures that require bone exposure), smoking, chemotherapy, use of corticosteroids, and periodontitis [95,96,97].

Clazakizumab (NCT03744910), Gevokizumab (NCT00998699, NCT01788033), Mavrilimumab (NCT01706926), and Gimsilumab (NCT04205851) are monoclonal antibodies that have not been FDA-approved to treat any conditions, but they either have been or are currently being investigated to treat kidney transplant rejection, type I and II diabetes, rheumatoid arthritis, and ankylosing spondylitis, respectively. The three remaining drugs are inhibitors that block cytokine signaling. Interferon beta-1b (Betaseron/Extavia) is a recombinant human interferon that binds to type I interferon receptors (IFNAR1 and IFNAR2) and is FDA-approved as an immunosuppressant to treat relapsing-remitting forms of multiple sclerosis [98,99]. It promotes the expression of anti-inflammatory cytokines, including IL-10 [100,101], and reduces the expression of inflammatory cytokines, including IL-17 [101,102]. TAK-242 (resatorvid) inhibits TLR4 signaling by binding to the intracellular domain of TLR4 and interfering with the interactions between adaptor proteins and the receptor, thus inhibiting the expression of inflammatory cytokines [103]. Resatorvid was investigated as a potential therapeutic to reduce mortality rates in patients with severe sepsis. However, it failed to reduce cytokine levels in treated patients with sepsis or reduce mortality rates significantly [104]. Though this drug failed to treat systemic sepsis, the administration of resatorvid in the periodontal pockets, which are colonized by pathogenic, gram-negative bacteria, may serve as a potential therapeutic to reduce chronic inflammation. Galunisertib is an oral small inhibitor molecule that blocks TGF-β receptor 1 kinase activity. In humans, Galunisertib has been investigated as a potential treatment for several cancers, including metastatic pancreatic cancer, colorectal cancer, prostate cancer, ovarian carcinosarcoma, breast cancer, and glioblastoma (clinicaltrials.org, accessed on 10 March 2022). Galunisertib is not FDA-approved, but its inhibition of TGF-β signaling may be effective in reducing inflammation in periodontal pockets.

The efficacy of these drugs in treating periodontitis has yet to be investigated. However, this analysis indicates that these drugs may serve as potential therapeutics, either individually or in combination, to treat inflammation in periodontitis gingival tissue. All of these drugs in some way mitigate the inflammatory response or bone resorption via their mechanism of action. As noted above, reducing the inflammation induced by periodontitis may also reduce the severity of other systemic diseases by reducing the number of inflammatory cytokines and activated immune cells that circulate or migrate to other tissues throughout the body. Thus, additional experimentation is justified to further investigate the efficacy of these drugs in reducing the inflammation in the oral cavity of animal models for periodontitis, such as ligature-induced periodontitis in mice.

## 4. Materials and Methods

### 4.1. RNA-seq Analysis

A total of 26 periodontal disease SRA files, which were comprised of 22 periodontitis RNA samples, as well as 22 healthy RNA samples, were downloaded from two series (GSE173082 and GSE80715) retrieved from NCBI Gene Expression Omnibus (GEO). The RNA samples from the GSE80715 series were isolated from nine periodontal-healthy patients (one patient donating two samples for a total of 10 healthy samples), four moderate-periodontitis patients (one patient donating two samples), and three severe-periodontitis patients (two patients donating two samples each for a sum of 10 periodontitis samples). Patient information about the RNA samples from the GSE173082 series was not provided by the investigators on NCBI GEO. Information on the titles of each series, platforms used, library construction, sample type, diagnostic criteria, sample prep, and PubMed ID are provided in Table 1. Periodontitis and healthy RNA samples were analyzed using the Snakemake-based ARMOR workflow within a dedicated Conda environment, as described by Orjuela [39]. Briefly, quality control was performed on all RNA reads using fastQC. Reads with sufficient quality scores were trimmed using TrimGalore prior to mapping and quantification to the human GRCh38 reference transcriptome using Salmon [40]. Transcript quantifications from Salmon were then summarized at the gene level quantifications prior to performing differential gene analysis using edgeR [41].

### 4.2. Signaling Pathway Analysis

All of the Ensembl Gene IDs from edgeR were converted into their corresponding NCBI Gene identifiers utilizing the BioMart database [105]. The NCBI Gene IDs with significant *p*-values (*p* < 0.05) as calculated by edgeR, together with their fold-change values, were then used as the input for the signaling pathway impact analysis (SPIA) algorithm [42].

### 4.3. Drug Target Analysis

The SPIA output file was used as the input for drug target analysis which was performed using the Pathways2Targets R script that had previously been developed [46]. Briefly, identifiers from each of the pathways identified in the SPIA output file were retrieved and converted into UniProt identifiers. These protein identifiers were then used to query the public OpenTargets.org database for known human drug targets and the drugs that affect those targets [47].

### 4.4. Protein-Protein Interactions Analysis

Drug targets were used to construct the PPI network using the Search Tool for the Retrieval of Interacting Genes (STRING) [43] database (Version 11.5, http://string-db.org/, accessed on 7 March 2022). The PPI network was visualized in the Cytoscape [44] software (Version 3.9.0), and cytoHubba (a Cytoscape plugin for ranking nodes in a network by their network features) (Version 0.1; Chung-Yen Lin et al., Taipei, Taiwan) [45], and MCODE plugins were used to calculate the degrees of nodes and to identify significant modules. The top 10 transcripts with the highest degrees were identified as hub genes.

## Figures and Tables

**Table 1:** Summary of two individual public datasets of periodontitis.

GEO Gene Set ID	GSE173082	GSE80715
Title	Differential DNA methylation and mRNA expression in gingival tissues in periodontal health and disease	Transcriptome analysis of chronic periodontitis patients’ gingival tissue
Platform	Illumina HiSeq 4000	Illumina HiSeq 2000
Library Construction Protocol	Gingival tissue samples were harvested in conjunction with an invasive oral surgical procedure required for the participant’s oral care after administration of local anesthesia. Poly-A pull-down was carried out to enrich mRNAs from total RNA samples (200 ng–1 g per sample) followed by library preparation using the Illumina TruSeq RNA prep kit.	Frozen tissues were disrupted in the lysis solution of mirVana RNA isolation kit (Thermo Fisher Scientific) using disposable pestle grinder system (Thermo Fisher Scientific). After purification of mRNA molecules by poly-T oligo-attached magnetic beads followed by fragmentation, the RNA of approximately 300 bp size was isolated using gel electrophoresis. The cDNA synthesis and library construction were performed using the Illumina Truseq RNA sample preparation kit (Illumina, San Diego, CA, USA) following the manufacturer’s protocol.
Sample Type	Single End	Paired End
Diagnostic criteria	Not recorded	On the basis of clinical and radiographic criteria, periodontitis-affected site had a probing depth of ≥4 mm, clinical attachment level of ≥4 mm, and bleeding on probing.
Sample Prep	Not recorded	The size of 3 mm^2^ gingival biopsies were obtained from the marginal gingiva during periodontal flap surgery and immediately stored in RNAlater solution (Thermo Fisher Scientific, Waltham, MA, USA) at −70 °C after removal of blood by brief washing in phosphate-buffered saline.
Number of healthy samples vs. periodontitis samples	12 vs. 12	10 vs. 10
Number of healthy patients	Not recorded	nine periodontal healthy patients with pocket depth < 4 mm
Number of periodontitis patients	Not recorded	four periodontitis patients with pocket depth of 4–6 mm; three severe periodontitis patients with pocket depth of 7 mm or deeper
PubMed ID	Not published	27531006 [48]

**Table 2:** Top 22 DEGs identified in periodontal disease gingival tissue.

	Ensembl Gene ID	Symbol	Description	logFC *	logCPM **	*p*-Value	FDR ***
1	ENSG00000099958	DERL3	Derlin 3	3.92	4.40	3.45 × 10^−5^	2.92 × 10^−2^
2	ENSG00000170476	MZB1	Marginal zone B and B1 cell specific protein	3.98	5.29	4.48 × 10^−5^	2.92 × 10^−2^
3	ENSG00000153208	MERTK	MER proto-oncogene, tyrosine kinase	1.58	1.32	4.56 × 10^−5^	2.92 × 10^−2^
4	ENSG00000183508	TENT5C	Terminal nucleotidyltransferase 5C	3.11	5.28	6.44 × 10^−5^	2.98 × 10^−2^
5	ENSG00000198794	SCAMP5	Secretory carrier membrane protein 5	2.67	2.54	6.79 × 10^−5^	3.03 × 10^−2^
6	ENSG00000137265	IRF4	Interferon regulatory factor 4	3.14	4.02	7.33 × 10^−5^	3.03 × 10^−2^
7	ENSG00000061656	SPAG4	Sperm associated antigen 4	3.28	1.63	7.87 × 10^−5^	3.03 × 10^−2^
8	ENSG00000112936	C7	Complement C7	2.46	−0.06	8.31 × 10^−5^	3.03 × 10^−2^
9	ENSG00000100219	XBP1	X-box binding protein 1	1.81	7.87	8.99 × 10^−5^	3.05 × 10^−2^
10	ENSG00000065413	ANKRD44	Ankyrin repeat domain 44	1.41	3.31	9.72 × 10^−5^	3.05 × 10^−2^
11	ENSG00000117322	CR2	Complement C3d receptor 2	5.14	0.83	1.19 × 10^−4^	3.39 × 10^−2^
12	ENSG00000189233	NUGGC	Nuclear GTPase, germinal center associated	2.02	0.63	1.25 × 10^−4^	3.48 × 10^−2^
13	ENSG00000134285	FKBP11	FKBP prolyl isomerase 11	2.02	4.53	1.34 × 10^−4^	3.57 × 10^−2^
14	ENSG00000102096	PIM2	Pim-2 proto-oncogene, serine/threonine kinase	2.60	4.33	1.58 × 10^−4^	3.94 × 10^−2^
15	ENSG00000198018	ENTPD7	Ectonucleoside triphosphate diphosphohydrolase 7	0.70	4.47	1.70 × 10^−4^	4.15 × 10^−2^
16	ENSG00000130768	SMPDL3B	Sphingomyelin phosphodiesterase acid like 3B	1.99	1.39	1.76 × 10^−4^	4.20 × 10^−2^
17	ENSG00000101194	SLC17A9	Solute carrier family 17 member 9	2.46	1.75	1.84 × 10^−4^	4.25 × 10^−2^
18	ENSG00000153162	BMP6	Bone morphogenetic protein 6	1.89	2.26	1.95 × 10^−4^	4.30 × 10^−2^
19	ENSG00000073849	ST6GAL1	ST6 beta-galactoside alpha-2,6-sialyltransferase 1	2.12	5.13	1.97 × 10^−4^	4.30 × 10^−2^
20	ENSG00000198854	C1orf68	Chromosome 1 open reading frame 68	−3.78	1.50	2.03 × 10^−4^	4.36 × 10^−2^
21	ENSG00000122188	LAX1	Lymphocyte transmembrane adaptor 1	2.77	2.44	2.33 × 10^−4^	4.81 × 10^−2^
22	ENSG00000091490	SEL1L3	SEL1L family member 3	2.15	4.70	2.45 × 10^−4^	4.92 × 10^−2^

* logFC: Log_2_ fold change (log_2_FC); metric used to quantify the magnitude and direction of gene expression change (i.e., a gene is up- or downregulated in periodontitis samples compared to healthy samples). Positive values indicate upregulated genes and negative values indicate downregulated genes. ** logCPM: log counts per million; metric used to normalize read counts per gene after the read mapping process to enable the identification of significant changes in gene expression. *** FDR: false discovery rate-adjusted *p*-value.

**Table 3:** Signaling pathways identified as significantly impacted in periodontal diseased gingival tissue by SPIA.

	Name	pSize	NDE	pNDE	tA	pPERT	pG	pGFdr	pGFWER	Status	SourceDB
1	Cytokine–cytokine receptor interaction	177	39	1.27 × 10^−5^	13.49	1.20 × 10^−3^	2.90 × 10^−7^	5.01 × 10^−5^	5.01 × 10^−5^	Activated	KEGG
2	Staphylococcus aureus infection	29	13	3.66 × 10^−6^	9.22	1.55 × 10^−1^	8.72 × 10^−6^	7.55 × 10^−4^	1.51 × 10^−3^	Activated	KEGG
3	Natural killer cell-mediated cytotoxicity	95	23	1.70 × 10^−4^	47.51	1.28 × 10^−2^	3.06 × 10^−5^	1.47 × 10^−3^	5.29 × 10^−3^	Activated	KEGG
4	Chemokine signaling pathway	157	30	1.52 × 10^−3^	31.64	1.60 × 10^−3^	3.39 × 10^−5^	1.47 × 10^−3^	5.87 × 10^−3^	Activated	KEGG
5	Osteoclast differentiation	108	25	1.94 × 10^−4^	13.41	6.88 × 10^−2^	1.63 × 10^−4^	5.65 × 10^−3^	2.83 × 10^−2^	Activated	KEGG
6	Leukocyte transendothelial migration	76	19	3.94 × 10^−4^	19.35	4.92 × 10^−2^	2.30 × 10^−4^	6.63 × 10^−3^	3.98 × 10^−2^	Activated	KEGG
7	Keratinization	90	27	6.00 × 10^−7^	−1.90	2.00 × 10^−1^	2.03 × 10^−6^	1.43 × 10^−3^	1.43 × 10^−3^	Inhibited	Reactome
8	Innate Immune System	633	101	4.65 × 10^−5^	67.93	7.80 × 10^−2^	4.90 × 10^−5^	1.01 × 10^−2^	3.47 × 10^−2^	Activated	Reactome
9	Assembly of collagen fibrils and other multimeric structures	45	12	2.51 × 10^−3^	7.31	1.60 × 10^−3^	5.38 × 10^−5^	1.01 × 10^−2^	3.80 × 10^−2^	Activated	Reactome
10	Formation of the cornified envelope	62	19	1.96 × 10^−5^	−1.89	2.18 × 10^−1^	5.71 × 10^−5^	1.01 × 10^−2^	4.03 × 10^−2^	Inhibited	Reactome

pSize: the number of nodes in the pathway. NDE: number of differentially expressed genes based on unadjusted *p*-value. PNDE: hypergeometric *p*-value for enriched DEGs in pathway. tA: total net accumulated perturbation (tA). pPERT: bootstrap *p*-value. pG: unadjusted global probability. pGFdr: FDR correction of pG *p* < 0.05. pGFWER: Bonferroni-corrected pG. Activated/Inhibited: predicted effect on signaling pathway based on the direction of the tA value.

**Table 4:** List of top 10 drug targets.

	Target Symbol	Target Name(s)	Drug ID	Drug Name	Approved by FDA	Highest Clinical Trial Phase	Health Condition Investigated
1	IL6R; IL6ST	Interleukin 6 receptor; Interleukin 6 cytokine family signal transducer	CHEMBL3833307	Satralizumab	TRUE	4	AQP4 antibody-positive Neuromyelitis optica spectrum disorder (NMOSD)
2	TNFSF11	TNF superfamily member 11 (RANKL)	CHEMBL1237023	Denosumab	TRUE	4	Postmenopausal osteoporosis
3	IFNAR2	Interferon alpha and beta receptor subunit 2	CHEMBL1201563	Interferon Beta-1B	TRUE	4	Relapsing-remitting forms of multiple sclerosis
4	IL17RA	Interleukin 17 receptor A	CHEMBL1742996	Brodalumab	TRUE	4	Moderate to severe plaque psoriasis
5	TLR4	Toll-like receptor 4	CHEMBL225157	Resatorvid	FALSE	3	Severe sepsis
6	IL6	Interleukin 6	CHEMBL2108589	Clazakizumab	FALSE	3	Kidney failure, antibody-mediated rejection of kidney transplants, rheumatoid arthritis, asthma, Crohn’s disease, psoriatic arthritis, and COVID-19.
7	IL1B	Interleukin 1 beta	CHEMBL1743026	Gevokizumab	FALSE	3	Scleritis, colon cancer, osteoarthritis, chronic uveitis, Pyoderma Gangrenosum, gastroesophageal cancer, renal cell carcinoma, rheumatoid arthritis, Muckle–Wells syndrome, Behcet’s disease, and Type I and Type II Diabetes
8	TGFBR1	Transforming growth factor beta receptor 1	CHEMBL2364611	Galunisertib	FALSE	2	Metastatic pancreatic cancer, colorectal cancer, advanced hepatocellular carcinoma, prostate cancer, ovarian carcinosarcoma, rectal adenocarcinoma, breast cancer, nasopharyngeal cancer, and glioblastoma
9	CSF2RB	Colony stimulating factor 2 receptor subunit beta	CHEMBL1743039	Mavrilimumab	FALSE	2	Rheumatoid arthritis; acute respiratory failure and hyperinflammation in COVID-19
10	CSF2	Colony stimulating factor 2	CHEMBL2109430	Gimsilumab	FALSE	2	Ankylosing spondylitis; COVID-19

## Data Availability

Publicly available datasets were analyzed in this study. These data can be found here: NCBI GEO accession GSE173082 and GSE80715.

## Supplementary Materials

The following supporting information can be downloaded at: https://www.mdpi.com/article/10.3390/ijms23105580/s1.
